# Combining electrophysiology and optogenetics for functional screening of pyramidal neurons in the mouse prefrontal cortex

**DOI:** 10.1016/j.xpro.2021.100469

**Published:** 2021-04-15

**Authors:** Kenichiro Nagahama, Shuhei Fujino, Takaki Watanabe, Naofumi Uesaka, Masanobu Kano

**Affiliations:** 1 Department of Neurophysiology, Graduate School of Medicine, The University of Tokyo, Tokyo 113-0033, Japan; 2 International Research Center for Neurointelligence (WPI-IRCN), The University of Tokyo Institutes for Advanced Study (UTIAS), The University of Tokyo, Tokyo 113-0033, Japan; 3 Graduate School of Medical and Dental Sciences, Tokyo Medical and Dental University, Tokyo 113-8510, Japan

**Keywords:** High Throughput Screening, Microscopy, Neuroscience

## Abstract

Here, we present a comprehensive protocol to analyze the roles of disease-related genes in synaptic transmission. We have developed a pipeline of electrophysiological techniques and combined these with optogenetics in the medial prefrontal cortex of mice. This methodology provides a cost-effective, faster, and easier screening approach to elucidate functional aspects of single genes in several regions in the mouse brain such as a specific layer of the mPFC.

For complete details on the use and execution of this protocol, please refer to Nagahama et al. (2020) and Sacai et al. (2020).

## Highlights

• Protocol to investigate roles of single genes in synaptic transmission in the mPFC
• Construction and validation of miRNAs for KD of target single genes
• *In utero* transfection of KD-miRNAs into subsets of pyramidal neurons in the mPFC
• Functional and morphological analyses of synapses in mPFC neurons with or without KD

## Graphical abstract

**Figure:**
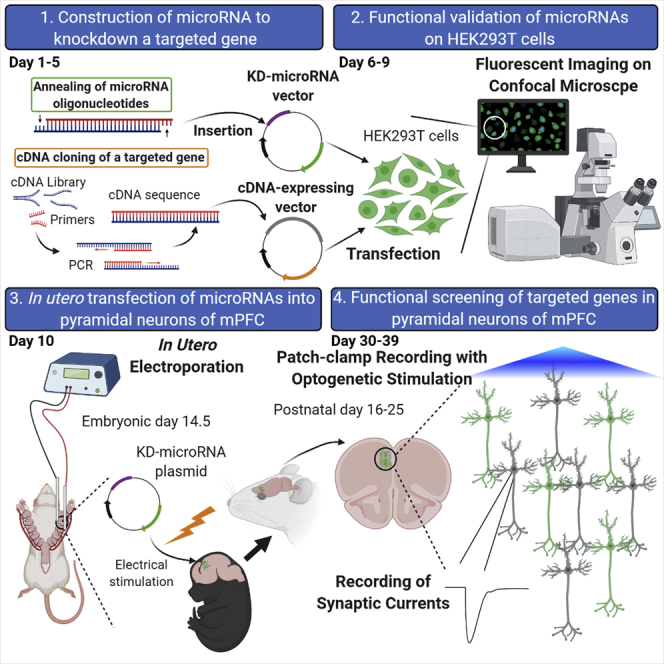

## Before you begin

### Design and generation of oligonucleotides for RNA interference (RNAi)-mediated knockdown experiment

**Timing: 3–5 days**

**Figure 1:**
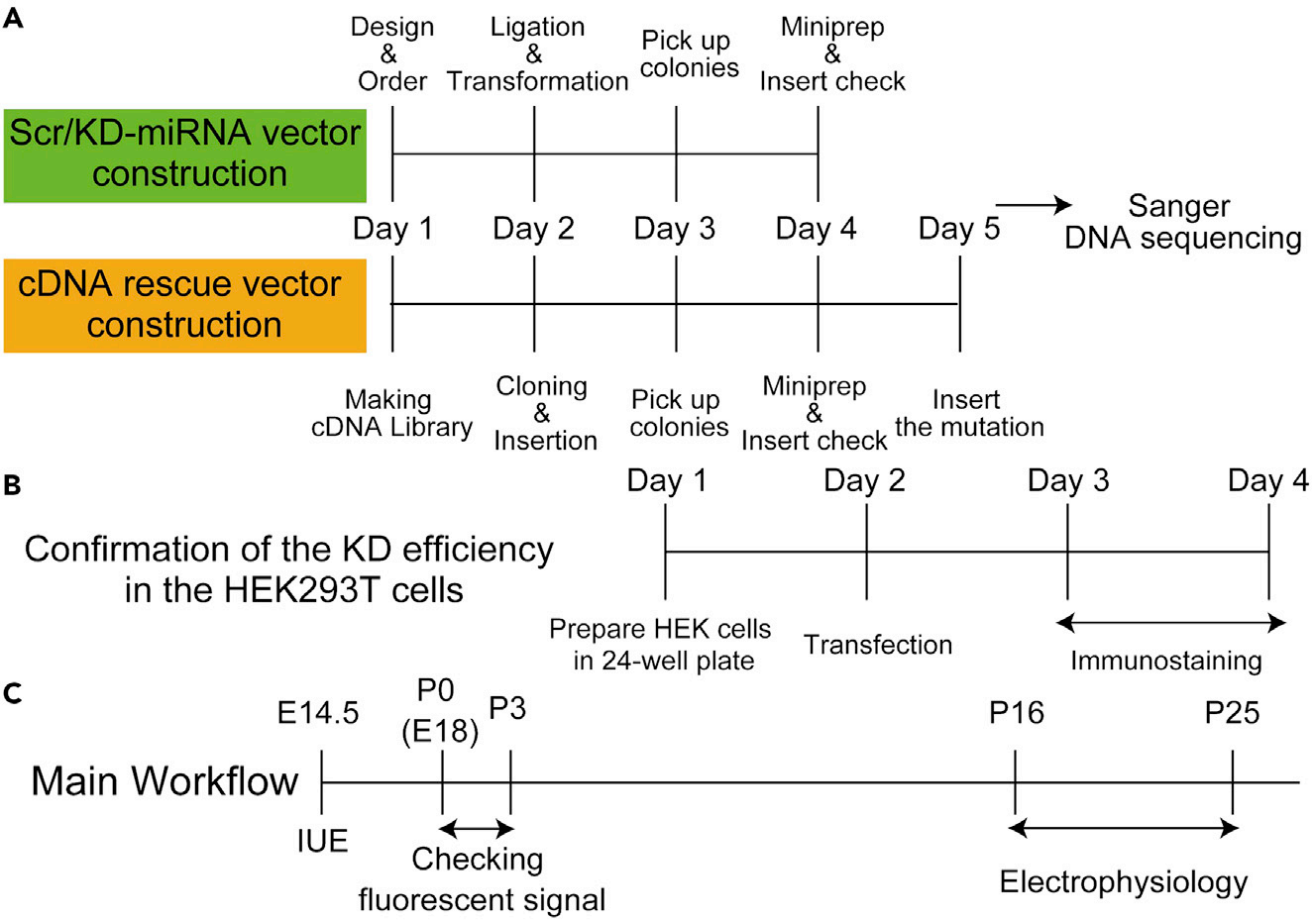
Workflow for the experiments in this protocol (A) Scheme of experimental time course for constructions of scramble (Scr) and knockdown (KD) vectors and cDNA-rescue vector. (B) Scheme of experimental time course for confirmation of KD efficiency of the KD vector in HEK293T cells. (C) Scheme of experimental time course for the main workflow from the *in utero* electroporation (IUE) to electrophysiological recordings.

**Figure 2:**
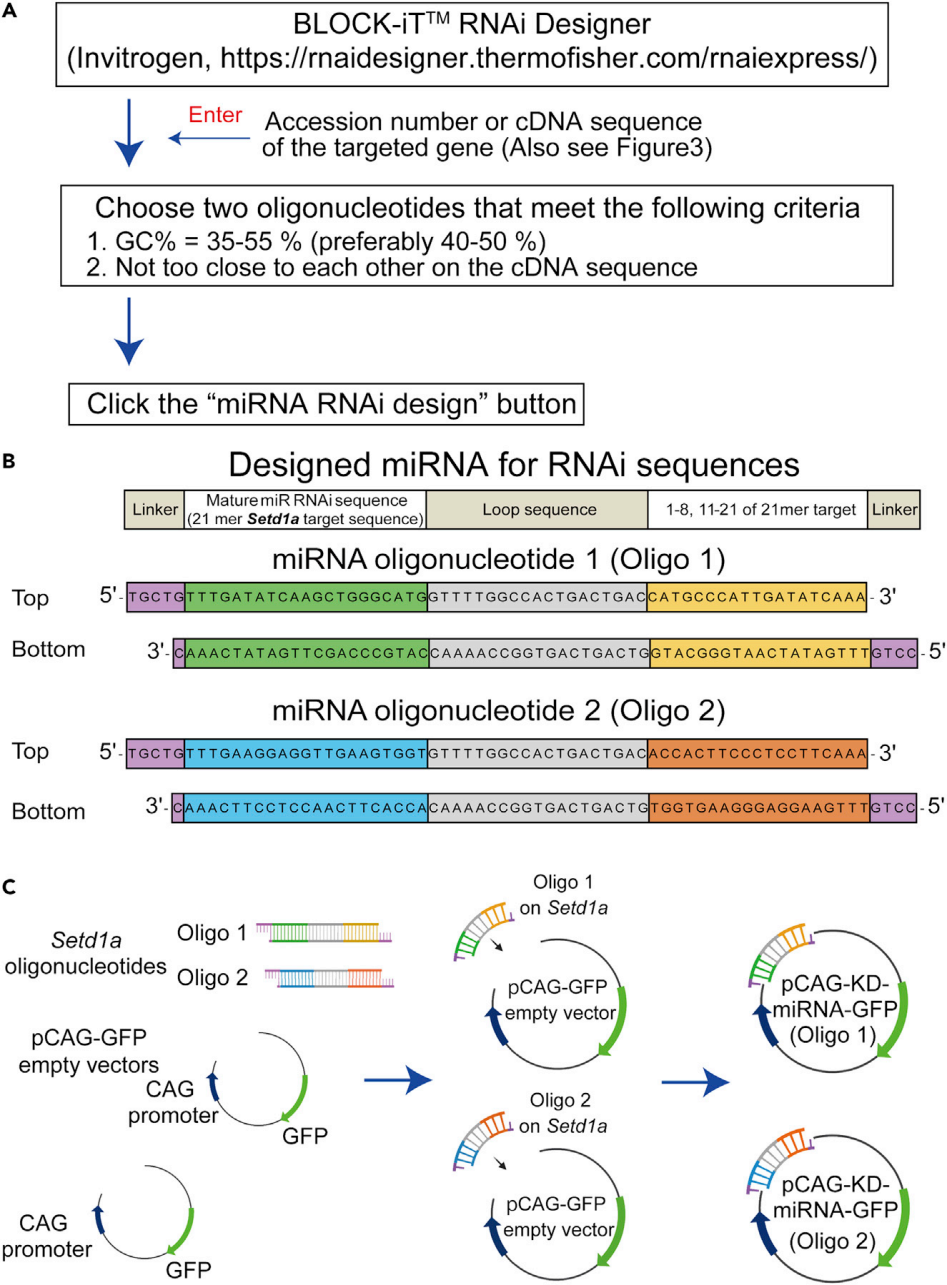
Scheme of workflow to construct oligonucleotides for miRNAs (A) Workflow for construction of miRNA oligonucleotides using the BLOCK-iT ™ RNAi Designer (Invitrogen, https://rnaidesigner.thermofisher.com/rnaiexpress/) website. Several 21-mer-oligonucleotide sequences are automatically provided depending on the specificity for sequences of targeted genes (the sequences with “5 stars” are recommended as highly specific sequences). (B) Scheme of the contents in the annealed miRNAs for the targeted gene (*Setd1a* as an example). The 64-mer oligonucleotides contain the linker sequence, the 21-mer sequence selected from the target gene, the loop sequence, and the 21-mer sequence excluding the 9th and 10th nucleotides. (C) Scheme of the process to insert the oligonucleotides into the pCAG-GFP empty vectors.

Choose specific sequences on the targeted disease-related genes using BLOCK-iT™ RNAi Designer (Invitrogen, https://rnaidesigner.thermofisher.com/rnaiexpress/) as an initial step of this protocol (Figure 1 and 2).

***Note:*** To exclude off-target effects, you should choose the sequences that the Invitrogen’s designer recommends. In our experiences, sequences on which the designer puts five stars would be effective to knock down targeted genes. See detail in the Invitrogen’s manual.

***Note:*** The two identical sequences should effectively decrease the expression of the targeted genes.

### Preparation of pCAG-GFP empty vector

***Alternatives:*** We used the pCAG-GFP (Addgene # 11150) to construct a pCAG-GFP vector containing a frame sequence for miRNAs (pCAG-GFP empty vector). Any other pCAG-GFP vectors will work as well.

1. Subclone a frame sequence for miRNAs (miR frame sequence) from pcDNA^TM^6.2-GW/EmGFP-miR included in the BLOCK-iT Pol II miR RNAi Expression Vector Kit with the following primers; forward: 5’-AAAGCGGCCGCCCTGGAGGCTTGCTGAAGG-3’, reverse: 5’-AGTGCGGCCGCGGCCATTTGTTCCATGTGA-3’ under the same PCR protocol as subcloning a cDNA sequence (See the Protocol No. 25-No. 34 at the step 1-2).
2. Linearize 1 μL pCAG-GFP (Addgene # 11150) plasmid with 1 μL Not1 in 18 μL reaction buffer at 37°C for 1 h.
3. Insert the PCR product containing the miR frame sequence into the linearized pCAG-GFP using the Ligation high Ver. 2 (See the Protocol No. 32 at the step 1-2).
4. Transform the product at 3. into DH5α (See Protocol No. 5-No. 19 at the step 1-1).
5. After getting the pCAG-GFP empty vector, linearize 1 μL solution containing pCAG-GFP with 1 μL Esp3I (BsmBI) and 2 μL Tango buffer in the 16 μL MilliQ water at 37°C for 12–24 h as a preparation to construct pCAG-miRNA-GFP.

***Note:*** To prevent self-ligation of the pCAG-GFP empty vector during long-term storage, we recommend to perform dephosphorylation of the vector. We usually use rAPid Alkaline Phosphatase kit (Roche Molecular System, #4898141001). Any other kits also work as well.

### Preparation of cDNA library

**Timing: 1 day**

We usually use the RNeasy Mini Kit (QIAGEN, USA) for the RNA extraction and PrimeScript^TM^ 1^st^ strand cDNA Synthesis kit (Takara Bio. USA) for reverse transcription from the RNA to cDNA. Also See each manufacturer’s instructions in detail. All of the buffers are included in the kit.

6. Cut the region of your interest (mPFC) of brain from the sacrificed mouse under anesthesia.
7. Measure weight of the tissue in a 1.5-mL plastic tube. The weight should be kept around 30 mg.
8. Apply the 600 μl lysis buffer (Buffer RLT in the kit) for homogenization to the tissue.
9. Disrupt and homogenize the tissue in the lysis buffer using glass homogenizer.
10. Put the lysate into a new 1.5-mL tube and centrifuge the lysate for 3 min at >13,000 × *g*.
11. Remove the supernatant of the lysate carefully from the tube and put it into the other new 1.5-mL tube.
12. Add 1-mL 70% ethanol to the supernatant of the lysate and mix by pipetting.
13. Apply the sample into a spin column with a 2-mL collection tube (included in the kit).
14. Centrifuge the sample for 30 s at >13,000 × *g* and discard the flow-through.
15. Add 700 μl stringent washing buffer (Buffer RW1 in the kit) to the column.
16. Centrifuge for 30 s at >13,000 × *g* and discard the flow-through.
17. Add 500 μl mild washing buffer (Buffer RPE in the kit) to the column
18. Centrifuge for 30 s at >13,000 × *g* and discard the flow-through.
19. Place the spine column including the RNA in a new 1.5-mL tube.
20. Add 40 μl RNase-free water directly to the inside of the column.
21. Centrifuge for 30 s at >13,000 × *g* and discard the flow-through.
22. Aliquot the sample to the several tubes as you want.

**Pause point:** The sample can be stored at –80°C for long-term keeping.

23. Mix the 1–2 μL RNA sample, 1 μL 6 mer primer, and 1 μL dNTP mixture and make it up to 10 μL with RNase-free water.
24. Incubate the mixed sample at 65°C for 5 min and transfer it on ice immediately.
25. Mix the 10 μL sample with 4 μL 5× buffer, 0.5 μL RNase inhibitor, and 1.0 μL PrimeScript RTase with 4.5 μL RNase-free water gently.
26. Incubate the sample at 30°C for 10 min and 42°C for 60 min.
27. Inactivate the reaction enzyme by incubating the sample at 95°C for 5 min after the reaction.
28. Transfer the sample on ice and keep the sample at –20°C for further experiments.

***Alternatives:*** To make the cDNA library, we use the RNeasy Mini Kit (QIAGEN, USA) and PrimeScript^TM^ 1^st^ strand cDNA Synthesis kit (Takara Bio. USA). Other analogous kits will also work to extract the RNA.

**CRITICAL:** We usually extract RNA from 3- to 4-week-old male mice because of the estrus cycle that may affect gene expression. The ideal age of the mice upon RNA extraction will vary by the gene of interest. If the targeted gene is prevalent in adults and/or female, older and/or female mice should be used.

29. Keep the cDNA library at −80°C for construction of the cDNA rescue vector.

## Key resources table

**Table:** 

REAGENT or RESOURCE	SOURCE	IDENTIFIER
**Antibodies**		

Rat anti-GFP	Nacalai Tesque	Cat: # 04404-84; RRID: AB_10013361
Rabbit anti-RFP	MBL	Cat: # PM005; RRID: AB_591279

**Chemicals, peptides, and recombinant proteins**		

VECTASHIELD® HardSet^TM^ Antifade Mounting Medium with DAPI	Merck Millipore	CAT: # H-1500-10
QuikChange Lightning Site-Directed Mutagenesis Kit	Agilent	CAT: # 210518
X-tremeGene^TM^ HP DNA Transfection Reagent	MilliporeSigma	CAT: # 6366244001
BLOCK-iT Pol II miR RNAi Expression Vector Kit	Invitrogen	CAT: # K493600
RNeasy Mini Kit	QIAGEN	CAT: # 74104
KOD-plus-Neo	Toyobo, Japan	CAT: # KOD-401
Wizard® Genomic DNA Purification Kit	Promega	CAT: # A1120
PrimeScript^TM^ 1^st^ strand cDNA Synthesis kit	Takara Bio USA Inc.	CAT: # 6110A
PureYield^TM^ Plasmid Miniprep System	Promega	CAT: # A1222
Ligation high Ver. 2	Takara Bio USA Inc.	CAT: # LGK-201
Gibco^TM^ OPTI-MEM® Reduced Serum Medium	Thermo Fisher Scientific	CAT: # 31985-063
Agar powder	Nacalai Tesque, Japan	CAT: # 01028-85
LB Broth, Lennox	Nacalai Tesque, Japan	CAT: # 20066-95
Ampicillin sodium	Nacalai Tesque, Japan	CAT: # 02739-32
2,3-Dihydroxy-6-nitro-7-sulfamoyl-benzo [f] quinoxaline (NBQX)	Tocris	CAT: # 0373
D-(-)-2-amino-5-phosphonopentanoic acid (D-AP5)	Tocris	CAT: # 0106
Tetrodotoxin (TTX)	Nacalai Tesque	CAT: # 32775-51
Picrotoxin (PTX)	Nacalai Tesque	CAT: # 28004-71
Esp3I (BsmBI)	Thermo Fisher Scientific	CAT: # ER0451
EcoRI	New England BioLabs, Inc	CAT: # R0101
AgeI	New England BioLabs, Inc	CAT: # R0552
MSCI	New England BioLabs, Inc	CAT: # R0534
rAPid Alkaline Phosphatase	Roche Molecular Systems, Inc	CAT: # 4898141001

**Biological samples**		

HEK293T cell line	ATCC	CAT: # CRL-3216; RRID: CVCL_0063

**Experimental models: organisms/strains**		

Mouse: Institute of Cancer Research (ICR) (embryonic day 14.5)	Charles River	n/a
DH5α	Takara Bio Inc.	CAT: # 9057

**Recombinant DNA**		

pCAG-GFP	Addgene	CAT: # 11150
pmOrange2-N1 Vector	Takara Bio USA, Inc	CAT: # 632549
pCAG-DIO-mOrange	n/a	n/a
pAAV-EFa-DIO-mVenus	n/a	n/a
pCAG-FLEX-rc[Chronos-GFP]	Addgene	CAT: # 59056
pCAG-Cre	Addgene	CAT: # 13775

**Software and algorithms**		

ImageJ	NIH	https://imagej.net/Downloads
PATCHMASTER	HEKA	https://www.heka.com/
FITMASTER	HEKA	https://www.heka.com/
Neurolucida 360	MBF Bioscience	https://www.mbfbioscience.com/neurolucida-360-studio-2019
EZR (Ver. 1.53)	Kanda, 2013	http://www.jichi.ac.jp/saitama-sct/SaitamaHP.files/statmedEN.html
GraphPad Prism	GraphPad Software Inc	http://www.sigmaplot.co.uk/products/sigmaplot/sigmaplot-details.php
BioRender	n/a	https://biorender.com/

**Others**		

CUY650P5 Tweezers with Variable Gap, 2 Round Plate Electrodes (5 mm diameter) (a forceps-shaped electrode)	Unique Medical Imada	CAT: # CUY650P5
CUY21 SC Square Wave Electroporator and C200 Foot Switch	NepaGene	CAT: # CUY21SC
Micro-injector Picospritzer-III with footswitcher	Harvard Apparatus	CAT: # 052-0500-900 and # 050-0000-801-1
VT-1200	Leica	CAT: # 1491200S001
Glass capillary for electrophysiology	Harvard Apparatus	CAT: # EC1-30-0062
Glass capillary for *in utero* electroporation	Harvard Apparatus	CAT: # GC150F-10
Flaming/Brown Micropipette Puller P-97	Sutter Instrument Co.	CAT: # SU-P-97
EPC-10 amplifier	HEKA Elektronik	CAT: # 89-5001
MultiClamp 700B Microelectrode Amplifier	Molecular Devices, CA, USA	n/a
Axon Digidata 1550B Low-Noise Data Acquisition System plus HumSilencer	Molecular Devices, CA, USA	n/a
pCLAMP 11	Molecular Devices, CA, USA	n/a
Olympus BX51WI microscope	Olympus	n/a
IR-1000 Camera	DAGE-MTI	n/a
PC-Monitor	DAGE-MTI	n/a
LEXLZ4-B High-Powered LED Illumination system	SciMedia	CAT: # LEX2-LZ4-B
TC-324B Temperature Controller	Warner Instrument Co.	CAT: # TC-324B
Nunc Multidish 24 Nunclon^TM^ Delta Surface	Thermo Fisher Scientific	CAT: # 142475
C-1000 Touch^TM^ Thermal Cycler with Dual 48/48 Fast Reaction Module (C-1000 Touch Thermal Cycler)	Bio-Rad Laboratories, Inc.	CAT: # 1851148

## Materials and equipment

***Alternatives:*** This protocol uses a Choline Cl-based cutting solution for the preparation of acute mPFC slices. For older mice (>P60), a potassium gluconate-based cutting solution is recommended (Hong et al., 2014).

***Alternatives:*** This protocol describes the construction of microRNA (miRNA) plasmids using the BLOCK-iT Pol II miR RNAi Expression Vector Kit (Invitrogen, CA, USA) (Figure 2). Other analogous kits can also be used to construct the miRNA plasmids.

***Alternatives:*** For *in utero* electroporation (IUE), a forceps-shaped electrode (CUY650P5, Unique Medical Imada, Aichi, Japan) works for the electrophysiological experiments. Other commercially available probes are also suitable for the IUE in this protocol.

***Alternatives:*** This protocol uses the EPC-10 amplifier, PATCHMASTER, and FITMASTER (HEKA Elektronik, Lambrecht/Pfalz, Germany) to record and analyze the synaptic currents with the Olympus BX51WI microscope (Olympus). Other amplifiers and programs, such as the MultiClamp 700B, pCLAMP, and Clampex (Molecular Devices, CA, USA), can also be used for the recordings and analyses. Other differential interference contrast (DIC) microscopes will work as well.

**Table:** Cutting solution for acute slices of prefrontal cortex (Choline Cl-based)

Reagent	Final concentration (mM)	Stock concentration	Add to 1L
**10× stock solution**			

Choline Cl	120 mM	1200 mM	167.54 g
NaHCO_3_	28 mM	280 mM	23.52 g
NaH_2_PO_4_	1.25 mM	12.5 mM	1.95
KCl	2 mM	20 mM	1.49 g
MilliQ water			1 L
**Total**			**1 L**

**1× cutting solution**			

10× Stock solution	n/a	n/a	50 mL
CaCl_2_・2H2O	1 mM	1 mM	0.5 mL
MgCl_2_・6H2O	8 mM	1 mM	4 mL
Glucose	25 mM	n/a	2.25 g
MilliQ water			440 mL
**Total**			**500 mL**

Store at 4°C. Bubble 95% O_2_ and 5% CO_2_ gas through a thin tube for 30 min to 1 h before cutting acute slices.

**Table:** Artificial cerebrospinal fluid (ACSF) (Choo et al., 2017; Uesaka et al., 2012, 2018)

Reagent (stock solution #1)	Final concentration (mM)	Stock concentration	Add to 1L
NaCl	125 mM	1250 mM	73.05 g
KCl	2.5 mM	25 mM	1.86 g
NaH_2_PO_4_・2H_2_O	1.25 mM	12.5 mM	1.95 g
NaHCO_3_	26 mM	260 mM	21.84 g

**Reagent (stock solution #2)**	**Final concentration**	**Stock concentration (1 mM)**	**Add to 500 mL**

CaCl_2_・2H_2_O	2 mM	1 mM	147.02 mg

**Reagent (stock solution #3)**			

MgSO_4_・7H_2_O	1 mM	1 mM	123.24 mg
Stock Solution #1	n/a	n/a	50 mL
Stock Solution #2	n/a	n/a	1 mL
Stock Solution #3	n/a	n/a	0.5 mL
glucose	20	n/a	1801.06 g
MilliQ water			440 mL
**Total**			500 mL

Store at 4°C. Bubble with 95% O_2_ and 5% CO_2_ gas through a thin tube for 30 min to 1 h before cutting acute slices.

**Table:** Internal Solution in the patch pipette to record spontaneous EPSC (sEPSC), miniature EPSC (mEPSC), and intrinsic excitability (Maejima et al., 2005).

Reagent	Final concentration (mM)	Add to 50 mL MilliQ water
K D-gluconate	130 mM	1522.625 mg
KCl	6 mM	22.365 mg
NaCl	10 mM	29.22 mg
EGTA	0.5 mM	9.50875 mg
CaCl_2_	0.16 mM	1.17616 mg
MgCl_2_	2 mM	20.33 mg
HEPES	0.5 mM	119.155 mg
Na_2_-ATP	4 mM	110.22 mg
Na_2_-GTP	0.4 mM	11.3428 mg
pH 7.3, adjusted with KOH		
**Total**		**50 mL**

Store at −20°C.

**Table:** Internal Solution in the patch pipette to record spontaneous IPSC (sIPSC) and miniature IPSC (mIPSC) (Ade et al., 2008).

Reagent	Final concentration (mM)	Add to 50 mL MilliQ water
KCl	145 mM	540.4875 mg
HEPES	10 mM	119.155 mg
EGTA	10 mM	190.175 mg
CaCl_2_	0.16 mM	1.17616 mg
MgCl_2_	2 mM	20.33 mg
Mg-ATP	5 mM	136.3675 mg
Na_2_-GTP	0.2 mM	5.6714 mg
pH 7.2, adjusted with KOH		
**Total**		**50 mL**

Store at −20°C.

**Table:** Internal Solution in the patch pipette to record evoked synaptic responses electrically and optogenetically (Ding et al., 2012).

Reagent	Final concentration (mM)	Add to 50 mL MilliQ water
CsMeSO_3_	120 mM	1368 mg
CsCl	15 mM	126.27 mg
NaCl	8 mM	23.376 mg
EGTA	0.2 mM	3.8035 mg
HEPES	10 mM	119.15 mg
TEA-Cl	10 mM	82.855 mg
Mg-ATP	4 mM	50.718 mg
Na_2_-GTP	0.3 mM	8.5071 mg
Spermine	0.1 mM	1.74095 mg
QX314	5 mM	50 mg
pH 7.3, adjusted by CsOH		
**Total**		**50 mL**

Store at −20°C.

***Note:*** Parameters for the recording of evoked synaptic responses include the amplitude of evoked postsynaptic currents to assess the input-output relation, paired pulse ratio (PPR), and inhibition/excitation balance.

**CRITICAL:** Make the cutting solution and the ACSF approximately 30 min–1 h before the experiment. We do not recommend keeping the ACSF for more than 1 week. The stock solution of the ACSF should be used within 1 month. The internal solutions can be kept at −20°C for 6 months to 1 year.

## Step-by-step method details

### Major step 1-1: Construction of plasmid expressing RNA interference (RNAi)-mediated single gene knockdown and scramble vectors

**Timing: 3–5 days**

As a first step, you will make the following three different types of plasmids; 1) Control miRNA, 2) Scramble (Scr) miRNA, and 3) Knockdown (KD) miRNA for the targeted gene in which you are interested (Figure 1 and Table 1). For construction of the miRNA plasmids, we usually use BLOCK-iT Pol II miR RNAi Expression Vector Kit (Invitrogen) which includes the reagents that are required for the construction including a competent cell and Super Optimal Broth with Catabolite repression (S.O.C. medium). The following plasmids will be constructed for this protocol;

**Table 1:** Summary of plasmids in this protocol

Plasmid	Promoter	Reporter	Vector	Insert
pCAG-miRNA-GFP (Scr/KD miRNA)	CAG	GFP	pCAG-GFP vector (Control miRNA)	Scr/KD-miRNA oligonucleotide sequence
pCAG-miRNA-mOrange	CAG	mOrange	pCAG-mOrange vector	KD-miRNA oligonucleotide sequence
pCAG-WT-cDNA-mOrange	CAG	mOrange	pCAG-mOrange vector	native cDNA sequence
pCAG-cDNA rescue-mOrange	CAG	mOrange	pCAG-mOrange vector	cDNA sequence with base substitutions on the miRNA-binding sites
pCAG-DIO-mOrange	CAG	mOrange	pCAG-GFP vector	DIO: subcloned from pAAV-EFa-DIO-mVenus mOrange: subcloned from pmOrange2-N1 Vector (Takara Bio USA)
pCAG-DIO-Chronos-GFP	CAG	GFP	Purchased from Addgene (# 59056)	
pCAG-Cre	CAG	n/a	Purchased from Addgene (# 13775)	

To exclude possible side effects of GFP expression, we use an empty vector carrying only GFP without miRNA sequences as the control miRNA vector for the validation of KD efficacy of the miRNAs in HEK293T cells later.

***Note:*** The construction of pCAG-miRNA-mOrange is the same with that of pCAG-miRNA-GFP except the fluorescent proteins. The following process presents the protocol for the pCAG-miRNA-GFP. In the case of the pCAG-miRNA-mOrange, you will use pCAG-mOrange empty vector instead of the pCAG-GFP empty vector.

***Alternatives:*** We subcloned the mOrange sequence from pmOrange2-N1 Vector (Takara Bio USA) and replaced GFP with mOrange on the pCAG-GFP empty vector. Any other red fluorescent protein like tdTomato will work for constructions of the WT-cDNA and cDNA-rescue vectors, pCAG-DIO vector, and pCAG-miRNA vector as well.

***Alternatives:*** We subcloned the double-floxed inverted open reading frame (DIO) sequence from the pAAV-EFa-DIO-mVenus (gifted from Dr. Akiko Hayashi-Takagi) and replaced GFP by the DIO and mOrange sequence on the pCAG-GFP empty vector to construct the pCAG-DIO-mOrange. A DIO sequence from any other plasmids containing the DIO will work as well. We also recommend to replace the Chronos-GFP sequence on the pCAG-DIO-Chronos-GFP with the mOrange subcloned from the pmOrange2-N1 Vector.

Day 1

1. Mix 5 μL each of the top- and bottom-oligonucleotides constructed in the step “Design and Generation of Oligonucleotides for RNA Interference (RNAi)-Mediated Knockdown Experiment”. Add 1 μL of any kind of oligo annealing buffer.
2. Incubate the mixture at 84°C for 4 min.
3. Elute 1 μL of the annealed oligonucleotides with 499 μL MilliQ water. You can keep the leftover of the eluted oligonucleotides at −20°C.

***Note:*** In the meantime, thaw the ligation enzyme and competent cells (DH5α) on ice.

4. Mix 1 μL of the 500×-eluted oligonucleotides and 0.5 μL pCAG-GFP empty vector without any inserted miRNA oligonucleotides with 1.5 μL Ligation high Ver. 2. at 24°C–27°C for 1h. You can keep the leftover of the diluted oligonucleotides at −20°C.
5. Transform all of these samples into 50 μL of competent cells.
6. Incubate the sample on ice for 30 min.
7. Heat shock the sample at 42°C for 30 s.
8. Incubate the sample on ice for 2 min.
9. Add warm 250 μL S.O.C. medium to the sample.
10. Incubate it at 37°C for 45–60 min.
11. Spread the sample evenly on an LB agar plate with ampicillin.

***Note:*** We usually make the LB agar plate using agar powder (CAT: # 01028-85, Nacalai Tesque, Japan), LB medium powder (LB Broth, Lennox, CAT: # 20066-95, Nacalai Tesque, Japan) and ampicillin sodium (CAT: # 02739-32, Nacalai Tesque, Japan). We recommend 1.5% agar, 2% LB and 0.01% ampicillin should be included in the plate.

***Alternatives:*** The commercially-available LB plate can work as well.

12. Incubate the plate for 12–16 h at 37°C.

Day 2

13. Pick several colonies from the LB plate using plastic tips.
14. Put each tip into a tube (15 mL centrifuge tube) of 3 mL 2% LB medium with 300 μg ampicillin sodium.
15. Incubate the tubes for 12–16 h at 37°C.

Day 3

16. Use 1 mL of the 3 mL LB medium with the competent cells to make a glycerol stock for long-term storage at −80°C.
17. Use what is leftover of the 3 mL LB medium and competent cell mixture for mini-prep.
18. Perform the mini-prep following the manufacturer’s instructions.

***Note:*** We use PureYieldTM Plasmid Miniprep System (Promega, USA). Other miniprep kits work as well.

19. After the mini-prep, check the concentration of the plasmid. 300–450 ng/μL is a usual range of the concentration.

***Note:*** You can check the insertion of the oligonucleotides using restriction enzymes if the vector has the appropriate cleavage sites. It is better to confirm the insertion of the correct oligonucleotides by Sanger DNA sequencing before *in utero* electroporation.

***Note:*** In the BLOCK-iT Pol II miR RNAi Expression Vector Kit, we usually use MscI (NEB to confirm the insertion of the oligonucleotides of miRNA sequences.

### Major step 1-2: Construct a rescue vector to express cDNA of the targeted genes

**Timing: 1 week: Constructing a wild-type (WT)-cDNA vector**

**Timing: 3–5 days: Designing and Making a cDNA Rescue Vector**

For the rescue experiments, we will generate a vector expressing a miRNA-resistant cDNA of the targeted gene.

***Alternatives:*** We usually use KOD-plus Neo DNA polymerase to clone a sequence of the targeted gene from the cDNA library. The accuracy of this polymerase is quite good, but other polymerases may work as well.

**Figure 3:**
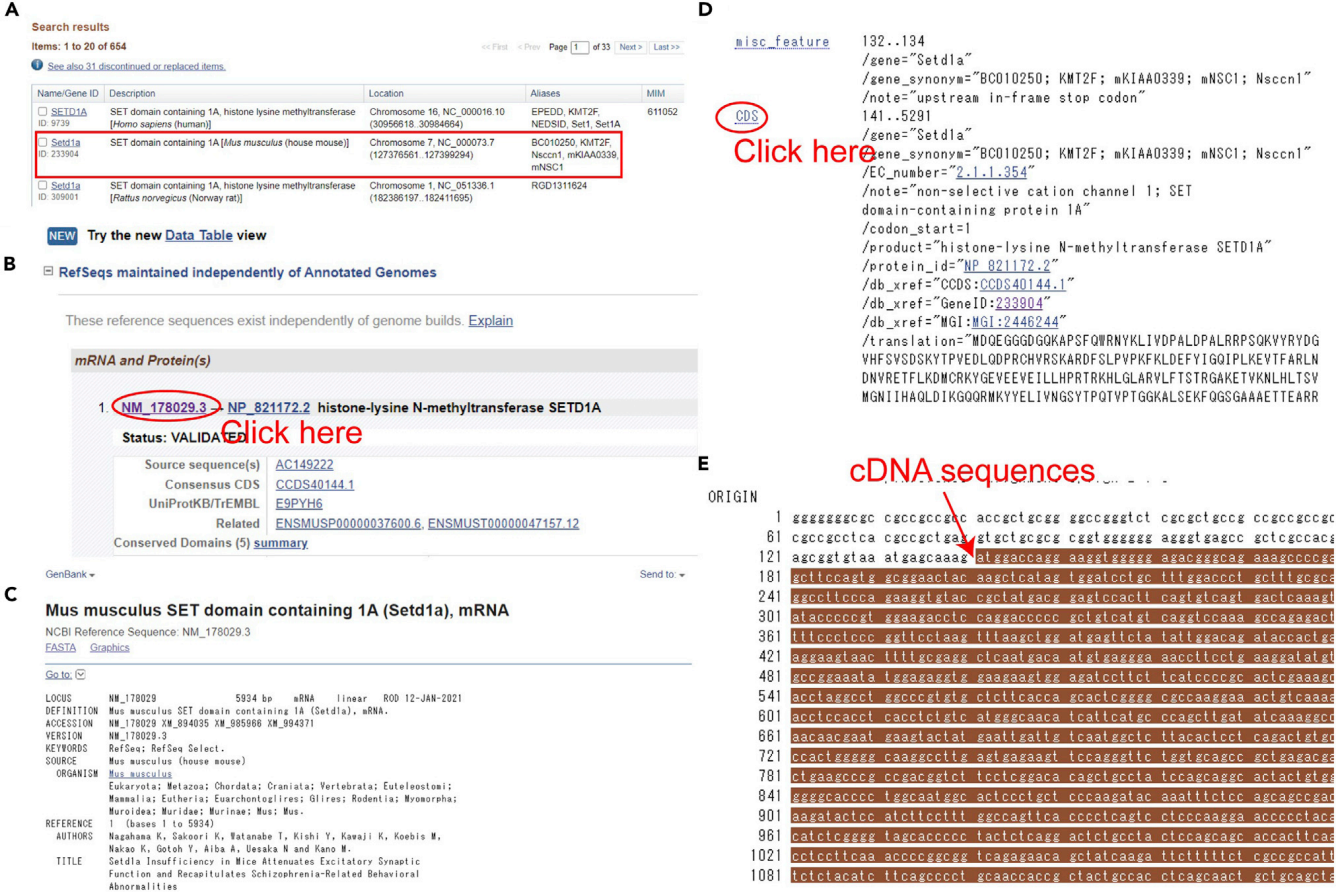
Process to search cDNA sequences in the NCBI database (A) Example of the genomic data of targeted gene (*Setd1a*) on different species. (B) mRNA and protein data of the mouse SETD1A protein. (C–E) A whole DNA sequence of *Setd1a* including the cDNA (C). Clicking the “CDS” tab (D) visualize the cDNA sequence of the targeted gene (E).
22. Click the mRNA tab on the “mRNA and Protein (s)” section (Figure 3B).
23. In the next page, click the “CDS” tab to highlight the coding region of the targeted gene (Figures 3C–3E).
24. Design both forward and reverse PCR primers for the cDNA to clone whole sequences of the targeted gene. Order the primers commercially.

***Note:*** We usually design PCR primers ourselves. A commercial website can also be used to design the primers.

20. Search the cDNA sequence of the targeted gene using the National Center for Biotechnology Information (NCBI) database (https://www.ncbi.nlm.nih.gov/).
21. Choose the gene name on *Mus musculus* (house mouse) from the search results (Figure 3A).

25. Use KOD-plus Neo DNA polymerase kit (TOYOBO, Japan) and make a pre-mixed master solution containing 4 μL 10× PCR buffer, 4 μL dNTPs, 2.4 μL MgSO_4_, 2.4 μL primer mix (1.2 μL forward and 1.2 μL reverse primers) (10 μM), 1.6 μL cDNA library that is already prepared at the “Preparation of cDNA Library” section, and 24.8 μL MilliQ water for each of the desired reactions. Vortex it well.
26. Add 0.8 μL of DNA polymerase per reaction to the pre-mixed master solution. Mix gently by pipetting.
27. Add 40 μL of the pre-mixed master solution into each 200 μL PCR tube.
28. Run the PCR using an appropriate program. One example of 2-step PCR is found as follows.

**Table:** Example of 2-step PCR protocol for cloning of the WT-cDNA sequence.

Step	Temperature	Time
1	95°C	10 min
2	98°C	10 s
3	62°C	30 s
4	68°C	30 s /kb
5	Go to step 2, 30× more times.	
5	68°C	7 min
6	4°C	∞

***Alternatives:*** We usually use C-1000 Touch Thermal Cycler (Bio-Rad, USA). Any other PCR machines work as well.

***Note:*** If the targeted gene is longer than 3.0 kb, we recommend dividing the sequence into at least 2 fragments. This is because the KOD-plus Neo DNA polymerase will only produce products up to 3.0 kb. Clone each fragment individually and ligate them prior to insertion into the pCAG vector (troubleshooting 1).

29. Perform 1.5% agarose gel electrophoresis using the PCR samples.
30. Extract the band from the agarose gel under UV light.
31. Purify the PCR product from the extracted band using the DNA purification kit.

***Alternatives:*** We usually use Wizard® Genomic DNA Purification Kit (Promega, USA). Other DNA purification kits will also work.

32. Mix 1 μL of the PCR product and 1 μL of linearized pCAG-mOrange empty vector without any inserted sequences with 2 μL of Ligation high Ver. 2 (Takara Bio USA, Inc., USA). Incubate this mixture at 24°C–27°C for 1 h.

***Note:*** Any other ligation enzyme can work as well.

***Note:*** We usually use GFP and mOrange as reporter markers for the miRNAs and cDNA respectively. Other fluorescent proteins work as reporters for the miRNAs and the cDNA if they can be distinguished visually.

33. Transform and isolate the plasmid as shown in the construction of the KD-miRNA plasmid (See the Protocol No. 5-No. 19 at the step 1-1).
34. Check the insertion of the cDNA sequence using restriction enzymes and agarose gel electrophoresis.

***Note:*** EcoRI and AgeI are effective at cutting the insert. Other restriction enzymes may work as well.

***Alternatives:*** We usually use the QuikChange Lightning Site-Directed Mutagenesis Kit (Agilent, CA, USA) to insert the mutations into the cDNA sequence. Any other mutation kit could work as well.

35. Design primers using the QuikChange Primer Design site (https://www.agilent.com/store/primerDesignProgram.jsp).

***Note:*** When designing the primers, replace 4–6 nucleotides in the miRNA-targeted sequences without changing any codons from the DNA sequences. It is critical to prevent binding of the KD miRNA to the cDNA rescue vector.

36. After getting the primers, mix 1 μL forward and 1 μL reverse primers (125 ng/μL each) with 1 μl WT-cDNA plasmid (50 ng/μL), 5 μl 10× reaction buffer, 1 μl dNTP mix, 1.5 μl QuikSolution reagent (included in the kit) and 38.5 μl MilliQ water in 200 μl PCR tubes. Vortex it well.
37. Add 1 μl QuikChange Lightning Enzyme into each PCR tube. Mix it gently by pipetting.
38. Run the PCR using appropriate program. One example of the program is found as follows.

**Table:** Example of 2-step PCR protocol for insertion of the mutation into the WT-cDNA vector.

Step	Temperature	Time
1	95°C	2 min
2	95°C	20 s
3	60°C	10 s
4	68°C	30 s /kb
5	Go to step 2, 30× more times.	
5	68°C	7 min
6	4°C	∞

39. After the PCR, apply 2 μl Dpn I restriction enzyme to the PCR mixed sample. Mix it gently by pipetting.
40. Incubate it at 37°C for 5 min.

Transform the sample into the competent cells in the same way as explained from the Protocol No. 5 to No. 19 in the step 1-1.

**CRITICAL:** All miRNA and cDNA expression vectors carrying fluorescent proteins (GFP or mOrange) should include the CAG promoter. The CAG promoter is most effective at inducing strong expression of the Scr/KD/cDNA vectors (Bitzenhofer et al.,2017). Other promoters, such as the EF1α and human synapsin promoters, do not induce efficient gene expression by *in utero* electroporation.

**CRITICAL:** The exact sequences of each plasmid should be confirmed by DNA sequencing.

### Major step 1-3: Evaluation of KD efficiency of the miRNAs on HEK293T cells

**Timing: 3 days**

**Figure 4:**
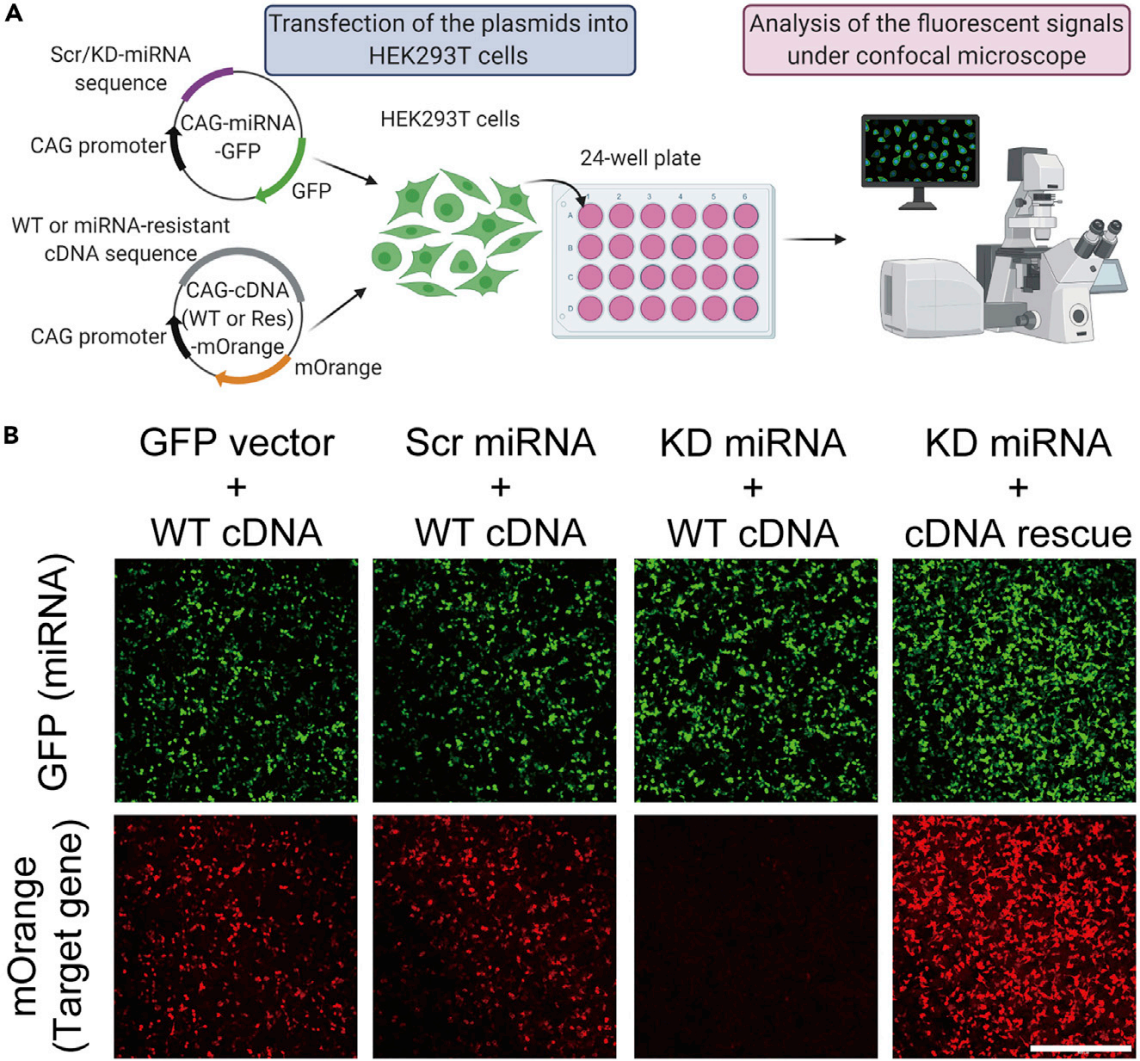
Validation of efficiency of the KD miRNA in HEK 293T cells (A) Illustration of workflow to evaluate the efficiency of the KD-miRNA vector compared to the Scr-miRNA vector for targeted genes in HEK293T cells. The illustration was made by BioRender. WT: wildtype, Res: rescue. (B) Representative images of the GFP and mOrange from the transfected HEK293T cells in each condition. Scale, 100 μm.

Validation of the miRNAs is necessary before applying them to *in vivo* experiments. We usually perform this validation in HEK293T cells (Figure 4A and 4B). Additionally, we recommend to quantify the mRNA expression by qPCR using the electroporated brain to confirm the reduction of the targeted gene (Nagahama et al., 2020).

Day 1

41. Prepare a 100 mm dish of 50% confluent HEK293T cells.
42. Replace the medium with HBSS (3 mL) and add trypsin (500 μL) to the new medium.
43. Incubate the dish for 3–5 min at 37°C with 95% O_2_ and 5% CO_2_ gas.
44. Add 7 mL HBSS to the dish and mix well.
45. Centrifuge the mixture in a 50 mL tube at 110 × *g* for 3 min at any temperature under 20°C.
46. Remove the supernatant and suspend the cells in 12 mL of DMEM medium.
47. Put 0.5 mL of the suspended cell solution into each well of a 24 well plate for cell culture (Nunc Multidish 24 Nunclon^TM^ Delta Surface).

Day 2

48. (6–24 h later) Prepare four 1.5 mL-tubes containing OPTI-MEM (50 μL/well; 300 μL/6 well).

***Note:*** 6 wells are enough to quantify the fluorescent signals in each condition.

49. Add mixed plasmids to the four 1.5-mL tubes for the following four different conditions:

**Table:** 

	WT cDNA + GFP vector	WT cDNA + KD miRNA	WT cDNA + Scr miRNA	cDNA rescue + KD miRNA
WT-cDNA (μg)	0.2	0.2	0.2	-
cDNA-rescue (μg)	-	-	-	0.2
GFP vector (control) (μg)	0.3	-	-	-
KD miRNA (μg)	-	0.3	-	0.3
Scr miRNA (μg)	-	-	0.3	-
Total (μg)	0.5	0.5	0.5	0.5

50. Add 1.5 μL of X-tremeGene^TM^ HP DNA Transfection Reagent (Millipore-Sigma) to each tube.
51. Keep the tubes at 24°C–27°C or 15 min.
52. Add the mixed solution to each well of the HEK 293T cells.
53. (6 h later) Repeat the same procedures from 8 to 12.

***Note:*** We recommend the repeated transfection of the vectors in the same HEK-cell wells because the mOrange signal of the cDNA vectors after the single transfection might not be strong enough to evaluate the KD efficiency.

***Note:*** It might be better to adjust the volume of the plasmids if the fluorescent signals are weak (troubleshooting 2).

Day 3

54. (24 h later) Remove the medium and add 4% paraformaldehyde (PFA) into each well.
55. Keep the plate at 24°C–27°C for 10 min.
56. Remove the PFA and wash three times using phosphate buffered saline (PBS).

**Stopping point:** The samples can be kept in 1-mL PBS at 4°C for several months.

57. Add 300 μL PBS containing 0.1% Triton X to each well. Keep at 24°C–27°C for 10 min.
58. Add 30 μL of donkey serum (as a blocking agent) to each well. Keep at 24°C–27°C for 30 min.
59. Wash the wells with PBS three times.
60. Add primary antibody for GFP (diluted 1:1000 with PBS) and RFP (diluted 1:300 with PBS).
61. Keep the samples at 4°C for 12–24 h.

***Note:*** It is possible to see the fluorescent signals from GFP and mOrange alone, but we recommend staining them by immunocytochemistry due to the weakness of their native signals.

***Note:*** The primary antibodies for the targeted proteins might be useful to visualize the presence of the molecule, if these antibodies are readily available.

Day 4

62. (24 h later) Wash the wells with PBS three times.
63. Incubate in the secondary antibody (diluted 1:400 with PBS) at 24°C–27°C for 1 hour.
64. Perform a confocal imaging of the fluorescent signals from each well.

***Note:*** To quantify the fluorescent signals, we recommend capturing 5-6 images per well.

***Alternatives:*** Any other fluorescent microscopes work for the quantification as well, but the quality of imaging is better in the confocal microscope depending on the sample condition.

### Major step 2: *In utero* electroporation

**Timing: 3–5 days**

You will transfect several types of combination composed of the different plasmids using the *in utero* electroporation (Table 2).

**Table 2:** Summary for combinations of electroporated plasmids

Combination of electroporated plasmids	Ratio of the plasmid volume	Purpose (experiment)
pCAG-miRNA-GFP (KD)	-	Any other experiments except the following contents
pCAG-miRNA-GFP (Scr/KD) pCAG-DIO-mOrange pCAG-Cre	pCAG-miRNA-GFP : pCAG-DIO-mOrange : pCAG-Cre = 2000 : 2000 : 1	Spine imaging & analyses
pCAG-miRNA-GFP (Scr/KD) pCAG-DIO-Chronos-GFP pCAG-Cre	pCAG-miRNA-GFP : pCAG-DIO-Chronos-GFP : pCAG-Cre = 1 : 1 : 1	Compare paired-pulse ration (PPR) between Ctr-Ctr and KD-Ctr pairs in Figure 6F
pCAG-miRNA-mOrange (KD) pCAG-DIO-Chronos-GFP pCAG-Cre	pCAG-miRNA-mOrange : pCAG-DIO-Chronos-GFP : pCAG-Cre = 2000 : 2000 : 1	Compare PPR and amplitude of synaptic responses between KD-Ctr and KD-KD pairs in Figure 6F

**Figure 5:**
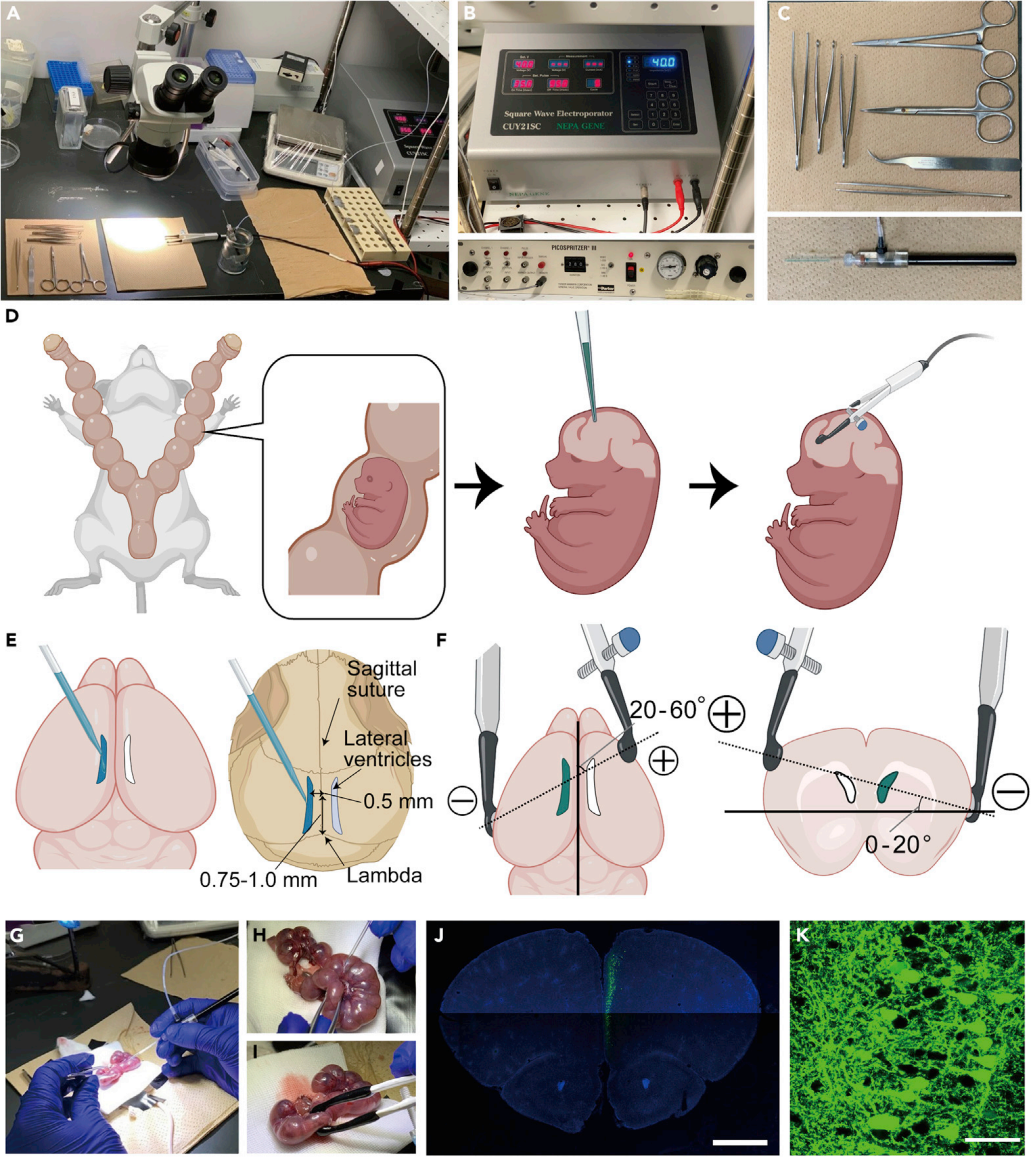
Experimental setup and essential equipment for *in utero* electroporation (A) Image of the whole setup for *in utero* electroporation. (B) Images of the electroporator to deliver electrical stimulation to pups (upper) and the micro injector to inject the plasmids (lower). (C) Images of surgical implements and a glass pipette which contains the diluted solution including plasmids for *in utero* electroporation. (D) Scheme of the *in utero* electroporation. The plasmid is injected into the fetal lateral ventricle and electrical stimulation is delivered via the forceps-shaped bipolar electrode. The electroporation is performed on 6–8 embryos in a single operation. (E) Inject the solution containing the plasmid into one side of the lateral ventricles on each embryo. The injection site of the plasmid should be 0.75–1.0 mm anterior from lambda and 0.5 mm lateral from the sagittal suture. (F) The positions of the electrode to target the mPFC. Note that the negative pole is positioned over the injected hemisphere while the positive one is positioned on the contralateral side. The two electrodes are slightly angled rostrocaudally and vertically as seen. (G–I) Low (G) and high (H) magnification Images of injection of the plasmids and image of delivering electrical stimulation through a forceps-shape electrode (I) during *in utero* electroporation. (J) Image of the mPFC from an electroporated mouse. The green signal shows GFP and the blue signal represents DAPI staining. Scale, 1mm. (K) High-magnification image of the electroporated mPFC expressing GFP. Scale, 50 μm. See also Methods video S1.

These steps induce efficient expression of the single gene-Scr/KD miRNA in layer 2/3 pyramidal neurons in the mPFC (Figure 5 and Methods video S1).

65. Prepare injection glass pipettes (1.5 OD × 0.86 IK × 100 L mm, # 30-0057, Harvard Apparatus, Co. UK) with a certain width (approximately 0.1–0.2 mm) of the tip right before the experiment using a micropipette puller (Flaming/Brown Micropipette Puller, # CAT SU-P-97, Sutter Instrument Co., USA). The pipettes are made by the following two-step puller protocol; Step1: HEAT=602, PULL=35, VEL=200, Time=250; Step2: HEAT 604, PULL=0, VEL=200, TIME=250.
66. Anesthetize a pregnant ICR mouse. Embryonic day 14.5 is suitable to target L2/3 pyramidal neurons.
67. Set the anesthetized pregnant mouse on a stage composed of several paper towels.
68. Open the abdominal wall of the dam and make 3–5 cm hole using a surgical scissors.
69. Pick up each pup with a round-shaped forceps gently from the hole on the abdomen.
70. Inject a 2–3 μL solution including a single plasmid or a combination of several plasmids into a lateral ventricle (Methods video S1). During the operation, you should keep the embryo wet using autoclaved PBS.
71. Set the bipolar electrode to target the mPFC.
72. Deliver electrical stimulations (35 V for 50 ms, 5 times at 950 ms intervals) via the forceps-shaped bipolar electrodes connected to the electroporator.
73. After finishing the electrical shock for the pups as you want, suture the abdominal hole using surgical strings and inject painkillers into the abdomen.

***Alternatives:*** Other hand-made bipolar electrodes such as a triple-electrode probe are also acceptable for *in utero* electroporation.

***Alternatives:*** The other strain such as C57B6 strain work as well for the *in utero* electroporation.

***Note:*** The concentration of the injected DNA plasmid is adjusted to 1.0–3.0 μg/μl with phosphate-buffered saline (PBS). The total volume of the injected plasmids is approximately 2–9 μg for each mouse.

***Note:*** To stimulate presynaptic neurons with miRNA-KD selectively and to compare the amplitudes and ratios of paired-pulse synaptic responses between the KD and control neurons in the same slices, you need to express an opsin sparsely in a subset of the KD neurons using Cre-loxP system. For example, we recommend transfecting 1–2 μg/μl of pCAG-DIO-Chronos-GFP plasmid and the same dose of the pCAG-KD-miRNA-mOrange plasmid with a smaller dose (0.5–1 ng/μl) of pCAG-Cre plasmid into L2/3 pyramidal neurons. These doses should be adjusted based on the expression levels of each fluorescent protein (Figure 8). Because the composition of opsin and fluorescent protein is possible to affect the function of the opsin, we have replaced GFP for mOrange as a reporter gene on the KD-miRNA plasmid for the sparse labeling of the Chronos (Figure 8).

**CRITICAL:** Use an extremely light-sensitive opsin such as Chronos because it is essential to increase the probability of obtaining the synaptic responses despite the opsin’s sparse expression. Other recent high-sensitive opsins will work as well.

74. 1–3 days after birth, verify the fluorescent expression in each pup through the skull with green/red-light glasses, and select the pups carrying efficient fluorescent expression.

***Note:*** Keeping only 6–7 pups might be appropriate for their proper development.

Methods video S1. Demonstration of *in utero* electroporation, related to Major step 2 and Figure 5

### Major Step 3: *Ex-vivo* electrophysiology on acute slices combined with optogenetics

**Timing: 4–5 h per day**

**Figure 6:**
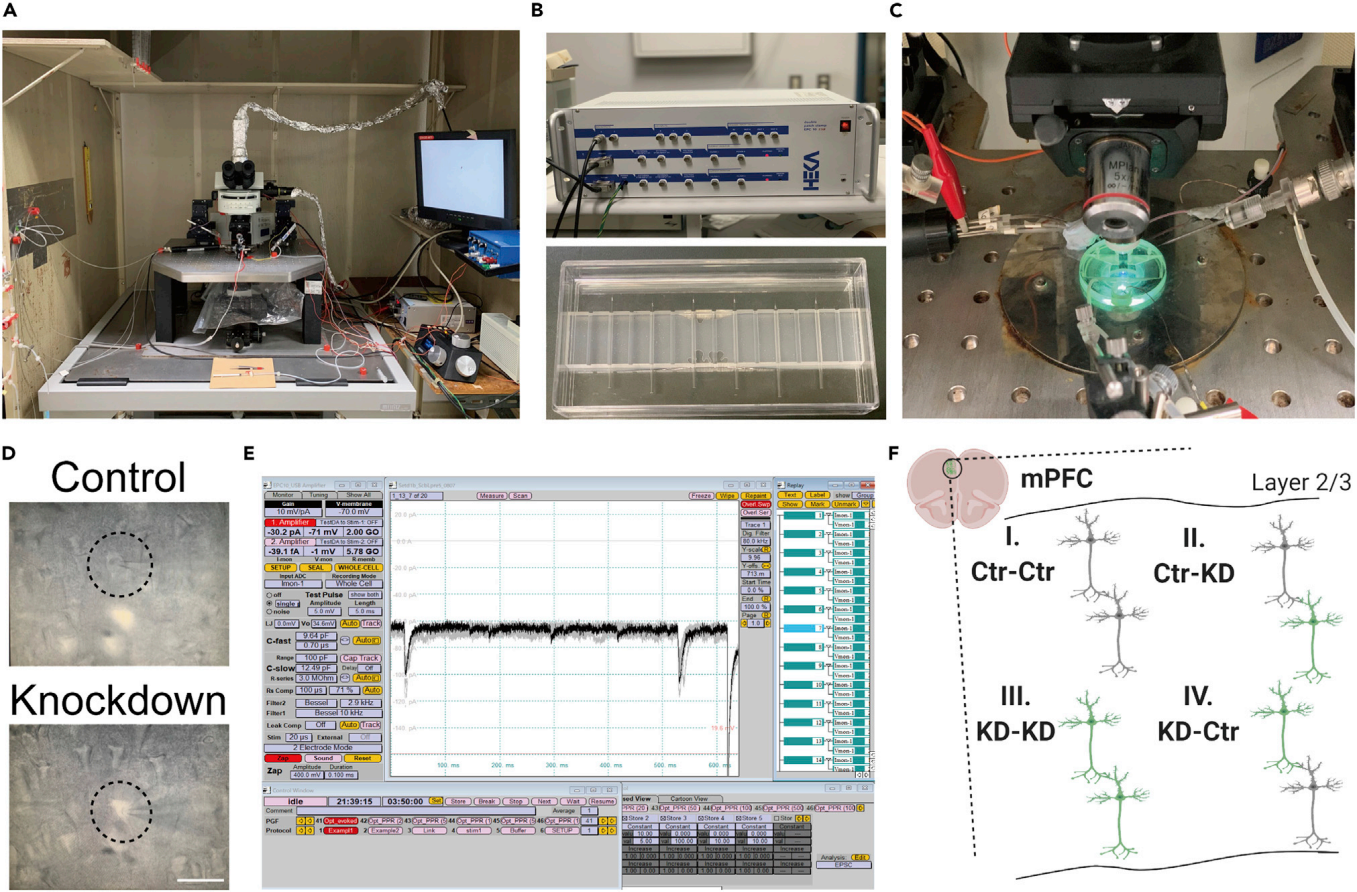
Experimental images from patch-clamp recording using LED light (A) Patch-clamp setup for electrophysiological experiments. (B) Amplifier for the recording (upper) and patch pipettes made from glass capillaries using a puller machine (lower). (C) Blue light shining during the experiments to confirm the fluorescent expression of mOrange or GFP. (D) Images of GFP-negative control and GFP-positive knockdown neurons through DIC microscope during the experiment. The upper neuron that is surrounded by the black circle is the control neuron and white cells mean the GFP-positive neurons (lower black circle). Scale, 50 μm. (E) PC screen on which the PATCHMASTER program is running to record synaptic currents evoked by optogenetic stimulation. (F) Scheme of possible neuron pairs in the electroporated mPFC.

***Note:*** The pipet resistance should be 2.4–5 MΩ for the layer 2/3 pyramidal neurons in the mPFC.

75. Prepare recording patch pipettes from borosilicate glass capillary (1.5 OD × 1.17 ID × 100 L mm, # 30-0062, Harvard Apparatus, Co. UK) using the Micropipette Puller (Model P-97, Sutter Instrument Co. USA). The 2-step protocols of the puller are; Step1, HEAT=572, PULL= 0, VEL=81, TIME=250; step 2, HEAT=570, PULL=0, VEL=60, TIME=250. It is recommended to adjust it appropriately according to the fineness of the pipette (Figure 6).

76. Chill the cutting solution on ice for 30 min–1 h bubbled with mixed gas composed of 95% O_2_ and 5% CO_2_.
77. Anesthetize and sacrifice a mouse with CO_2_ gas or another anesthetic in accordance with your approved Institutional Animal Care Use Committee (IACUC) protocol.

***Alternatives:*** Isoflurane also works well as an anesthetic.

***Note:*** It is easier to record synaptic currents in mice aged postnatal day 16–25 than in adult mice. For mice older than 1 month, we recommend cardiac perfusion of the Choline Cl-based cutting solution for 5–10 min prior to removing the brains from the mice.

We also recommend to use the potassium gluconate-based cutting solution for more than P60-older mice (Hong et al., 2014).

78. Remove the brain from the mouse immediately (< 1 min).
79. Put the brain into the cold cutting solution on ice. Incubate it around 3 min.
79. Trim the posterior part of the brain at the +1.0 to +2.0 mm anteriorly from the bregma.
80. Set the brain at the chamber of the slicer.
81. Start to cut the brain slices in the cold cutting solution at a certain thickness (see note below).
82. Cut 4–5 brain slices, including those from the mPFC.

***Note:*** The thickness of the slices depends on the brain region (e.g., Cerebral cortex: 300 μm; Cerebrum: 250 μm)

***Alternatives:*** This protocol uses a vibratome slicer (VT1200, Leica, Germany) to cut acute mPFC slices for electrophysiological recordings. Other slicers can be used as well.

83. Incubate the slices in a reservoir chamber at 24°C–27°C for 30–60 min with the ACSF.

***Alternatives:*** Incubation of the slices in a water bath at 37°C works as well.

84. Transfer one of the slices to the recording chamber on the stage of the differential interference contrast (DIC) microscope.
85. Place a patch pipette towards a targeted pyramidal neuron under the DIC microscope (Figure 6A-D). The temperature should be kept at 30°C–32°C during the recording. Otherwise, synaptic responses become small and it is hard to quantify them accurately.

***Note:*** For successful recording, we recommend choosing relatively small and teardrop-or pyramidal-shaped neurons in which you can clearly identify the soma (Figure 6D). Usually large neurons are either dead or severely damaged.

***Note:*** The layer 2/3 pyramidal neurons can be identified morphologically or by fluorescence. The neurons expressing GFP or RFP can be judged to be layer 2/3 pyramidal neurons because the layer showing the fluorescence is determined according to the date of the embryonic stages in which the *in utero* electroporation was performed (Fukuchi-Shimogori and Grove, 2001; Saito, 2006).

***Note:*** As far as we know, around 30% of the electroporated neurons are CaMKII-positive neurons in the mPFC (Nagahama et al., 2020; Sacai et al., 2020).

86. Put the patch pipette on the targeted neuron with positive pressure during approaching the neuron.
87. Release the positive pressure to make the tight seal between the pipette and the targeted neuron.

***Note:*** After putting the pipette on the neuron, you should change the holding potential from 0 mV to −70 mV gradually. The tight contact between the patch pipette and the membrane of the targeted neuron will be generated within several minutes, which is called a giga-ohm seal.

**CRITICAL:** Carefully watch how the cell responds to this activity. Sometimes rapidly decreasing the holding potential can be toxic to the targeted neuron.

88. Suck by mouth or make a negative pressure by a plastic syringe through tube connected to the glass pipette and connect the glass pipette to the targeted neuron (Figure 6D).

***Note:*** Small amount of electrical stimulation called as “Zap” sometimes work well to make the connection between the pipette and targeted neurons.

***Note:*** If the neuron expresses the GFP, you can see the fluorescent signal in the pipette after the suck.

89. After waiting about 5 min, record the synaptic current using stimulation protocols in the program using the amplifier (Figure 6E).

***Note:*** The synaptic current is recorded by the amplifier and filtered at a certain amount of the range. We usually use the default setting (2.9 kHz). The series resistance should be below 20 MΩ (preferably 10 MΩ or less) and must be controlled during the experiment.

***Note:*** Record the synaptic currents from the fluorescent-positive KD neurons or fluorescent-negative control neurons from the same electroporated mice (Figure 6D). Otherwise, it is also recommended to record and compare the currents from the fluorescent-positive Scr and KD pyramidal neurons in independent Scr and KD mice respectively.

***Note:*** Each parameter requires the presence of the following drugs in the perfused ACSF during the recording in each holding potential:

**Table:** 

Parameter	Drug	Holding potential
Spontaneous EPSC (sEPSC)	0.1 mM PTX	−70 mV
Spontaneous IPSC (sIPSC)	10 μM NBQX + 50 μM D-AP5	−70 mV
Miniature EPSC (mEPSC)	0.5 μM TTX + 0.1 mM PTX	−70 mV
Miniature IPSC (mIPSC)	0.5 μM TTX + 10 μM NBQX + 50 μM D-AP5	−70 mV
Evoked AMPAR-mediated EPSC	0.1 mM PTX (+50 μM D-AP5)	−70 mV
Evoked NMDAR-mediated EPSC	0.5 μM TTX + 10 μM NBQX	+40 mV
Evoked IPSC	10 μM NBQX + 50 μM D-AP5	0 mV

***Note:*** The appropriate holding potential should be changed depending on reversal potentials calculated by compositions of internal solutions and ACSF.

**CRITICAL:** Monitor the input resistance during the recording. Also, the value of the leak current could be critical in affecting the condition of the recording. We usually exclude the data showing the leak current under −200 pA.

90. Record the spontaneous and/or miniature synaptic currents initially to check the overall synaptic transmission in the targeted neurons.

***Note:*** If any abnormalities in either amplitude or frequency are found, we recommend recording the evoked synaptic responses by local electrical stimulation using a bipolar tungsten stimulating electrode as the next step. The magnitude of the amplitude and paired-pulse synaptic responses should be recorded (Nagahama et al., 2020; Sacai et al., 2020) (troubleshooting 3).

***Note:*** When applying the electrical stimulation, the bipolar tungsten stimulating electrode should be placed 50–100 μm away from recorded neurons to prevent the direct stimulation of neurons (Nagahama et al., 2020; Sacai et al., 2020).

**CRITICAL:** Keep the same distances between the electrode and a pair of the neurons with and without fluorescent proteins especially when analyzing the amplitude of the evoked synaptic responses because this directly affects the magnitude of the evoked synaptic responses.

***Note:*** When recording the paired-pulse synaptic responses induced by the electrical stimulation, it might be better to record them at several inter-stimulus intervals such as 20, 50, 100, and 500 msec (Nagahama et al., 2020; Sacai et al., 2020).

**Figure 7:**
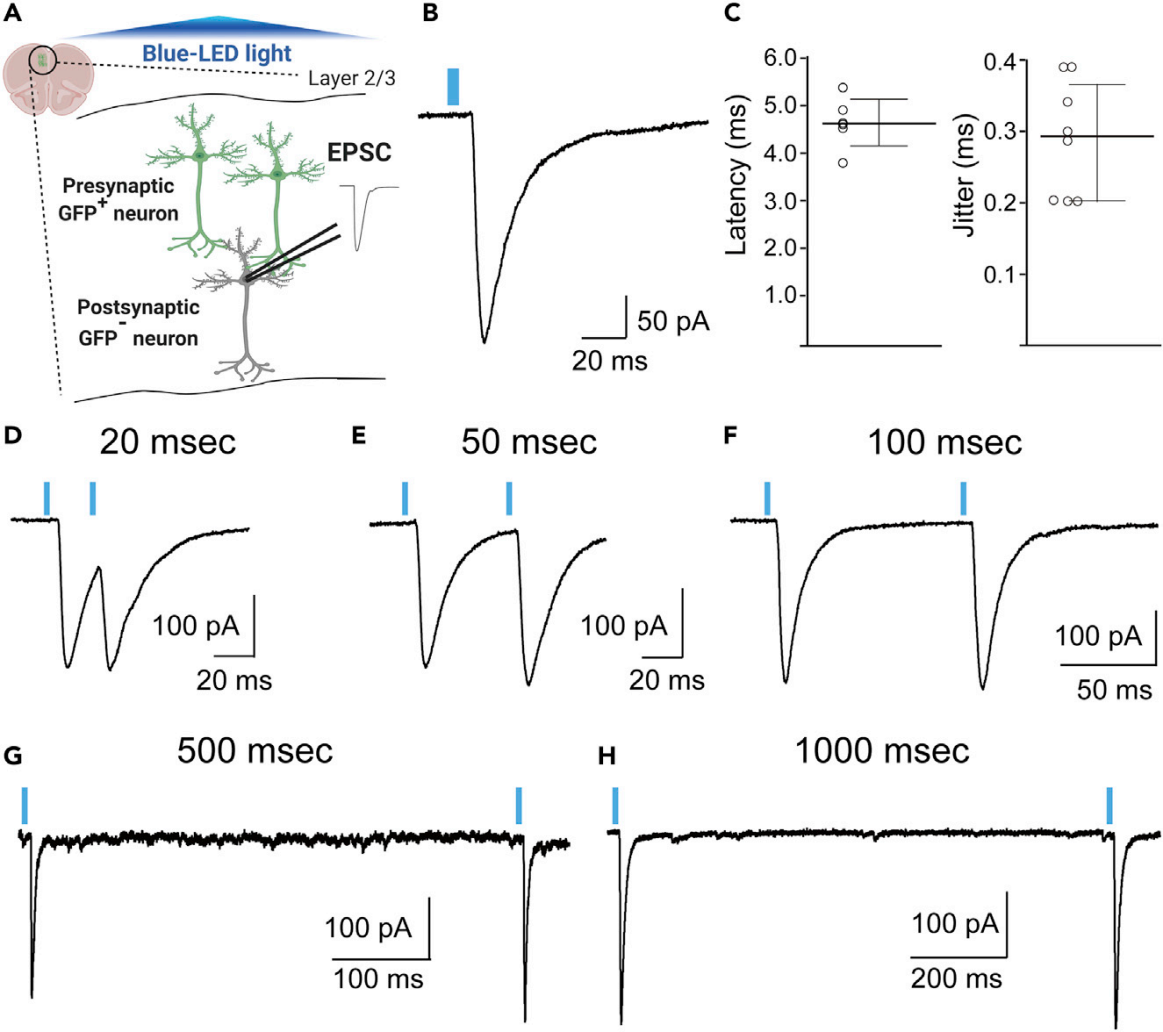
Optogenetically induced EPSCs from the fluorescent-negative neurons (A) Scheme of the patch-clamp recording from layer 2/3 (L2/3) pyramidal neurons in the mPFC combined with optogenetic light stimulation. Record from the GFP-negative control neurons. (B) Excitatory postsynaptic current induced by the activation of Chronos. (C) Latency (left) and jitter (right) of the optogenetically induced EPSCs. (D–H) Actual traces of the optogenetically induced paired-pulse EPSCs at 20 ms (D), 50 ms (E), 100 ms (F), 500 ms (G), and 1000 ms(H) inter-stimulus intervals. The paired-pulse responses were recorded in the presence of 100 μM picrotoxin.
  - To analyze effect of the KD in both pre- and postsynaptic neurons (Figure 6F; KD-KD and KD-Ctr), make the slices that both pre- and postsynaptic neurons show the KD miRNA expression and only presynaptic neurons show the opsin (Figures 8A–8D).
    a) Locate appropriate pairs of cells composed of the mOrange-positive KD and negative control pyramidal neurons without the opsin-GFP right next to the opsin-GFP/KD-miRNA-mOrange double positive pyramidal neuron (Figures 8A–8C).
    b) Perform whole-cell patch clamp recording on those pyramidal neurons one by one (Figure 8D).
    c) Apply paired-light-pulse stimuli at several inter-flash intervals (IFI). We usually use 20, 50, 100, 500 msec IFI (Figure 8E).
    d) Compare the amplitudes and the PPR values between non-fluorescent control neurons and the neurons showing the KD miRNA without the opsin in the same slices.
    ***Note:*** For this experiment, we used the pCAG-miRNA-mOrange to knock down the targeted gene because the pCAG-DIO-Chronos-GFP has the GFP expression under Cre recombination and we need to use the different reporter gene for the KD-miRNA expression.
    ***Note:*** 465-nm blue LED light pulse should be maintained at an appropriate strength (4 mW/mm^2^) and duration (2−10 ms) by the LED controller LEX2-LZ4-B (Brainvision, Inc., Tokyo, Japan). You can adjust these values to keep the value of the first amplitude in the PPR around 50−100 pA (Troubleshooting 4).
    ***Note:*** According to the shorter latency and the smaller jitter of the traces (Figures 7B and 7C), the Chronos-induced traces are monosynaptic responses. You can also validate the traces using TTX and 4-aminopyridine (4-AP) (Nagahama et al., 2020).

91. If any impairments in the initial paired-pulse ratio (PPR) are found, record the paired-pulse responses at 20, 50, 100, 500 ms inter-stimulus intervals (ISI) by the 0.1-ms electrical stimulation and 2 to 5-ms light shining to identify which pre or postsynaptic site is affected by the KD of targeted gene (Nagahama et al., 2020). There are four different types of expression patterns of the miRNAs in the pairs of pyramidal neurons in the electroporated mPFC (Figure 6F). In the first pair, both presynaptic and postsynaptic neurons are controls (Ctr-Ctr pair). The second pair means that the presynaptic neurons are control, but the postsynaptic neurons are GFP-positive KD neurons (Ctr-KD pair). The third one is a pair that both presynaptic and postsynaptic neurons are the GFP-positive KD neurons (KD-KD pair), and the fourth pair is composed of presynaptic GFP-positive KD neurons and postsynaptic GFP-negative control neurons (KD-Ctr pair) (Figure 6F and Table 3).
  - To analyze the effect of KD in only presynaptic neurons (Figure 6F; Ctr-Ctr and KD-Ctr), make the slices that only presynaptic pyramidal neurons should express the Chronos and the Scr/KD miRNAs with GFP (Figure 7A) from individual mice expressing Scr-miRNA or KD-miRNA plasmid respectively co-expressed with Chronos-GFP expression under Cre-induced recombination.
    a) Perform whole-cell patch clamp recording on the GFP-negative pyramidal neurons next to the opsin and GFP double positive pyramidal neuron (Figure 7A).
    b) Apply paired-light-pulse stimuli at several inter-flash intervals (IFI). We usually use 20, 50, 100, 500 msec IFI (Figures 7D–7G). You can also apply the light stimuli at longer IFI like 1,000 msec (Figure 7H).
    c) Compare the PPR values between the neurons from the mice showing the Scr miRNA + opsin and the KD miRNA + opsin.

**Figure 8:**
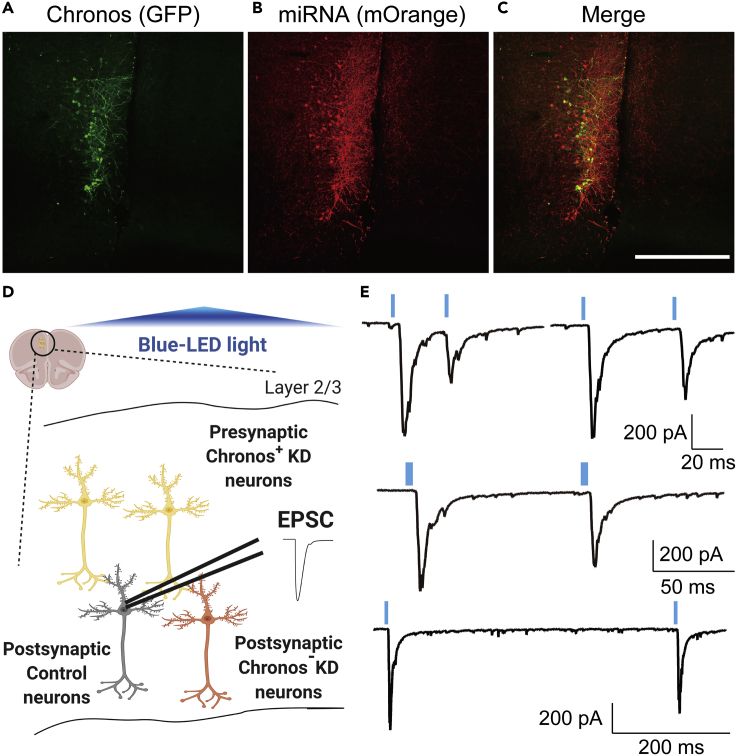
Sparsely-labeled opsin (Chronos with GFP) in the miRNA expressing neurons in the electroporated neurons (A–C) Representative Images of fluorescent expression of Chronos with GFP (A) and, KD miRNAs with mOrange (B), and the merge image showing the double-positive neurons (C). Scale, 500 μm. (D) Scheme of the patch-clamp recording using optogenetic stimulation. (E) Representative traces of optogenetically induced EPSCs from a non-fluorescent control neuron.
  - To analyze effect of the KD in only postsynaptic neurons (Figure 6F; Ctr-Ctr and Ctr-KD), you can stimulate presynaptic axons from layer 5/6 of the mPFC without any expression of the KD miRNAs (Nagahama et al., 2020).
    a) Set a bipolar tungsten stimulating electrode in the layer 5/6 without any fluorescent signals in the electroporated mPFC.
    b) Record paired-pulse-synaptic responses from appropriate pairs composed of the GFP-positive and negative pyramidal neurons in the layer 2/3 when stimulating the layer 5/6 by the electrode.
    c) Compare the amplitude and the PPR values between GFP-positive KD and negative control neurons.

**Table 3:** Summary for comparison of pairs of pyramidal neurons in paired-pulse stimulation

Comparison of cell pair in Figure 6F	Ratio of the plasmid volume	Combination of reporter gene	Stimulated-presynaptic neurons
Ctr-Ctr vs Ctr-KD	pCAG-miRNA-GFP (KD)	Presynaptic: Non-fluorescent Postsynaptic: GFP (KD) or non (Ctr)	Non-fluorescent layer 5 neurons by bipolar electrode
Ctr-Ctr vs KD-Ctr	pCAG-miRNA-GFP (Scr/KD): pCAG-DIO-Chronos-GFP : pCAG-Cre = 2000: 2000 : 1	Presynaptic: GFP (Scr miRNA or KD miRNA) Postsynaptic: Non-fluorescent	Scr or KD neurons expressing the Chronos by light shining (in individual mice)
KD-Ctr vs KD-KD	pCAG-miRNA-mOrange (KD): pCAG-DIO-Chronos-GFP : pCAG-Cre = 2000 : 2000 : 1	Presynaptic: GFP + mOrange (Chronos + KD miRNA) Postsynaptic: mOrange (KD) or non (Ctr)	mOrange-positive KD neurons expressing the Chronos by light shining

Also see Figure 6F.

***Note:*** Through these three different types of recording the paired-pulse responses, you can easily specify which synaptic site is responsible for the impairments of presynaptic glutamate release induced by KD of the targeted gene (Nagahama et al., 2020).

**Figure 9:**
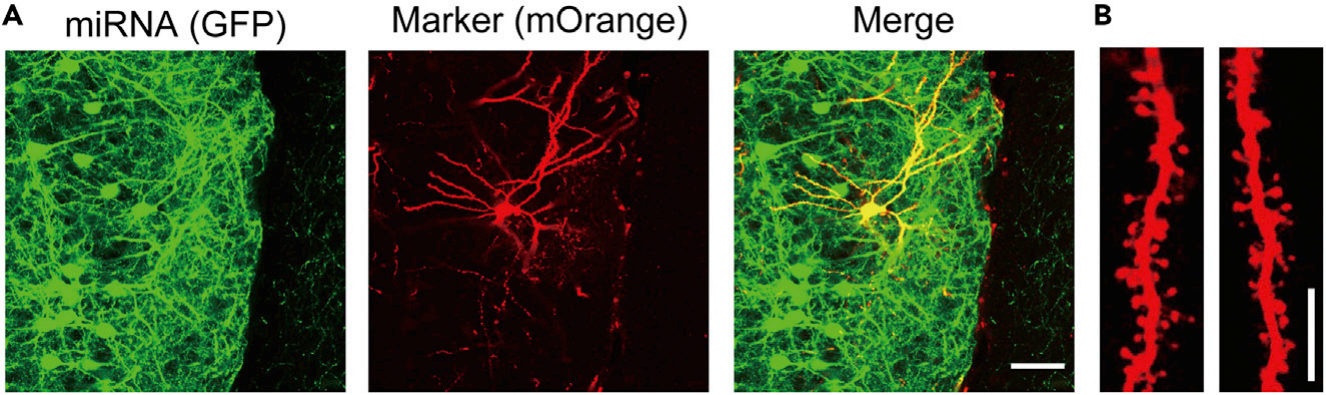
mOrange-sparse labeling for spine analysis (A) Images of the sparse labeling of GFP vector for the miRNA (left), mOrange vector (middle) as a reporter to label the individual neuron sparsely, and merge (right). Scale, 50 μm. (B) Representative images of dendrites from the mOrange-labeled neurons. Scale, 5 μm.

92. To analyze the KD effect on the postsynaptic site, perform immunohistostaining for GFP and mOrange in the slices and observe spine structures (Figure 9).
  a) Stain the slices, including the GFP-positive Scr/KD neurons from independent Scr/KD mice, using anti-GFP and RFP antibodies. Follow a general staining protocol.
  b) Observe the spine structures using a confocal microscope.
  c) Capture several images from each neuron (usually 5-6 images per neuron is optimal for quantification).
  d) Count the number of spines present and measure the head-width of each individual spine using a program such as Neurolucida360.
    ***Note:*** Using the mOrange signal expressed sparsely on the GFP-positive pyramidal neurons might be better to get more visible images in each spine. Alternatively, decreasing the volume of the injected miRNA-GFP vector would also be fine to express the GFP sparsely (troubleshooting 5).
    **CRITICAL: High-quality images are essential to analyze the spine structures. The criteria used to capture the images and detect each spine should be kept uniform for all neurons. We usually use the 60× oil immersion objective using digital zoom 2.0 at 0.48 μm-z series (1024 × 1024 pixels resolution) to capture the confocal images. To detect and classify the spines, we use the following criteria: outer range: 2.5 μm, minimum height: 0.3 μm, detector sensitivity: 50%, minimum count: 10 voxels for detecting spines and head-to-neck ratio: 1.1, length-to-head ratio: 2.5, mushroom head size: 0.35 μm, and filopodium length: 3 μm for the classification of spines** (Nagahama et al., 2020)**. Also see the instruction of Neurolucida 360.**
    ***Note:*** Additionally, you can record the evoked IPSC and evoked EPSC from the same pyramidal neuron to determine the inhibition/excitation balance. The following procedure can be a reliable way to record the inhibitory/excitatory (I/E) ratio (Nagahama et al., 2020)
  a) Record evoked EPSCs at the Cl^−^ equilibrium potential initially (−40 mV in this protocol) with the application of 50 μM D-AP5.
  b) Confirm the suppression of the evoked EPSCs by applying 10 μM NBQX to the bath solution.
  c) Record evoked IPSC at 0 mV, the reversal potential of EPSC in the protocol.
  d) Confirm the suppression of the evoked IPSC by 0.1 mM PTX.
    **CRITICAL:** The liquid junction potential calculated by the composition of the ACSF and each internal solution should be corrected.

## Expected outcomes

We clearly observe fluorescence expression in the layer 2/3 pyramidal neurons in the mPFC by *in utero* electroporation (Figure 5). Twenty to thirty percent of pyramidal neurons display fluorescence in the mPFC of the electroporated mice (Nagahama et al., 2020; Sacai et al., 2020). We can obtain the monosynaptic responses in each neuron using optogenetic-light stimulation (Figures 7 and 8).

We can also obtain the EPSC traces from each GFP-negative pyramidal neuron through patch-clamp recording. By combining the recording with optogenetics, we can manipulate the amplitude of paired-pulse synaptic responses by altering the strength and duration of the LED light. The first EPSC of the paired-pulse responses may have to be kept at 50–100 pA to analyze the PPR.

It is possible to simply compare the amplitude, the PPR, and the I/E ratio between the control and the KD neurons. This comparison allows us to evaluate the functional roles of disease-related genes expressed in pre- and postsynaptic neurons in synaptic transmission in the specific layer of the mPFC.

This protocol can provide faster timeline and easier manipulation of the genes because it just takes a couple of days to construct the plasmids and transfection into the brain. You can also work on several different genes as a functional screening on many candidates simultaneously once you get the miRNA-GFP or mOrange vectors for them.

To date, drugs or peptides which affect gene functions have been applied into bath solution or internal solution in a patch pipette to induce the gain- or loss-of-function of a target gene. However, spectrums of drugs are relatively limited, and these compounds may vary in their affinity to the target. Dominant-negative variants of a targeted gene which inhibit original functions of the gene can also be applicable to reducing the activity of the targeted gene. Although the *in utero* electroporation works for the transfection of the plasmids of those variants, it takes more time to construct the plasmids depending on the sequence of the targeted gene. Some of those plasmids are commercially available, but it is also limited to target only a couple of genes and it takes some additional costs. Compared to these conventional methodologies, our protocol enables to target most genes for which we can construct miRNA and cDNA rescue vectors.

To analyze how the targeted genes affect behavior, it is possible to combine the behavioral test battery with this protocol (Nagahama et al., 2020; Sacai et al., 2020).

## Quantification and statistical analysis

We usually use 3–5 mice per condition (Control or KD) for recording each parameter. Five to ten traces are enough to calculate the average values for a comparison of the input-output relation. For the PPR, we calculate the PPR values from 5–10 traces and average them in each interval. Twenty to thirty traces are averaged for the inhibition/excitation balance. The PATCHMASTER and FITMASTER programs (HEKA, Germany) can be used for the analyses of the input-output relationship, PPR, and inhibition/excitation balance. For the spine analyses, we usually capture 5-6 images from each neuron and average values of each parameter from 20 control and KD neurons respectively.

Mini Analysis Program (Synaptosoft) is useful to analyze the amplitude and the frequency of the spontaneous and miniature synaptic currents, and individual synaptic events can be detected by eye. Usually, we set the criteria to detect synaptic responses with an amplitude larger than 5 pA and with a rise time faster than 3 ms (troubleshooting 6).

***Note:*** It is also possible to create your own program to detect the individual synaptic responses.

For pairwise comparisons, Student’s *t* test or Mann-Whitney *U* test can be applicable depending on their distribution of data judged by the statistical analysis of normal distribution. For multiple comparisons, 2-way ANOVA will be suitable for the statistics.

***Note:*** We usually use EZR (Kanda, 2013) or Prism for the statistical analyses. The other statistical programs work as well.

## Limitations

Transfection using *in utero* electroporation is a common way to express the plasmid, but it has several limitations. First, the specificity of the brain region is relatively low because transfecting plasmids into the brain at high voltage induces the expression of the plasmids in layer 2/3 pyramidal neurons in not only the mPFC but also in other frontal cortical areas such as the primary and secondary motor cortices and orbitofrontal cortex (Nagahama et al., 2020; Sacai et al., 2020). The specific direction of the electrode during the *in utero* electroporation is the main strategy to control specificity of the brain region (Figure 5 and Methods video S1) (Taniguchi et al., 2012; Kamiya, 2009). As a second limitation, only a subset of the neurons can express the fluorescent signals; almost 20%–30% of the pyramidal neurons in the mPFC show the GFP expression (Nagahama et al., 2020; Sacai et al., 2020). Third, the expression of the plasmid is relatively low compared to gene expression induced by virus injection.

The whole-cell patch clamp recording also has several limitations when it is combined with optogenetics. As one limitation, the magnification of the amplitude of synaptic responses can be changed depending on the expression level of an opsin. Because of this limitation, we cannot compare the value of the EPSC amplitude between the individual animals showing the cell pairs composed of Scr miRNA + opsin or KD miRNA + opsin (Figure 7A). To compare the magnitude of the optogenetically-induced EPSCs between control and KD neurons, you can sparsely label the opsin and record the EPSCs from both types of neurons in the same slices by stimulating the opsin-positive presynaptic neurons (Figure 8). In addition, the value of the amplitude may decrease if less opsin is expressed. Sometimes we cannot obtain any synaptic responses for a whole day (troubleshooting 4).

## Troubleshooting

### Problem 1

It is difficult to find the appropriate restriction enzyme cleavage site(s) in the targeted gene sequence.

### Potential solution

In that case, we recommend using cloning techniques that do not require restriction enzymes and ligases. We usually use the In-Fusion® HD Cloning Kit (TaKaRa, Japan). Other similar cloning kits can work as well.

### Problem 2

Fluorescent signal of cDNA of the targeted gene is weak in the HEK293T cells.

### Potential solution

The volume of the cDNA plasmid should be increased. The volume of the KD miRNA should always be kept smaller than that of the cDNA plasmids, which makes it easy to see the reduced signal of the cDNA by the KD miRNA.

### Problem 3

It is difficult to obtain synaptic responses by local electrical stimulation.

### Potential solution

Adjust the position of the electrode and stimulate again. Sometimes the electrical conductivity of the electrode is critical to deliver sufficient stimuli to the slice. In that case, we recommend changing the electrodes.

### Problem 4

It is difficult to obtain synaptic responses by light stimulation.

### Potential solution

As mentioned in the “Limitations” section above, the amplitude of synaptic response depends on the expression level of the opsin. If the amplitude is not large enough to record paired-pulse synaptic responses, it might be better to increase the volume of the plasmid for expressing the opsin by *in utero* electroporation.

### Problem 5

It is difficult to locate sparse mOrange-labeled neurons in abundant GFP-positive KD neurons.

### Potential solution

This depends on the concentration of the floxed and Cre plasmid. If it is difficult to pipette the correct volume of the plasmid, we recommend increasing the number of trials of *in utero* electroporation performed.

### Problem 6

Signal-to-noise ratio of miniature EPSCs is low.

### Potential solution

Connect the electrical wires correctly and remove all unnecessary items around the electrophysiological setup. We recommend connecting all of the wires to one master power supply on the ground. Shielding the electrophysiological setup with the Faraday cage is likely to reduce noise from electrical devices on the outside of the cage. Turning off light might also work as well.

## Resource availability

### Lead contact

Further information and requests for resources and reagents should be directed to and will be fulfilled by the lead contact, Masanobu Kano (mkano-tky@m.u-tokyo.ac.jp).

### Materials availability

All of the plasmids generated in this study for *in utero* electroporation are available upon request.

### Data and code availability

No code is generated in this study.
